# Learning our lesson: using past policies to improve digital and ethnic inequalities beyond the pandemic

**DOI:** 10.1186/s13690-021-00744-8

**Published:** 2021-12-01

**Authors:** Mel Ramasawmy, Lydia Poole, Amitava Banerjee

**Affiliations:** Institute of Health Informatics, UCL, 222 Euston Road, NW1 2DA London, UK

**Keywords:** Digital health, Health inequalities, Digital divide, Health policy

## Abstract

COVID-19 has had a disproportionate impact on ethnic minorities in the UK, raising questions about whether learning from the past few decades about the interplay between ethnicity and health inequalities has been effectively incorporated in current health policy. As digital health approaches, such as remote consultations and apps, become more widespread during and after the pandemic, it is important to ensure that these do not contribute to ‘widening the gap’. We highlight three areas in which existing knowledge and evidence can be translated into cross-sectoral action to avoid further ethnic and digital health inequalities: data and measurement, improved communication, and embedded equality impact.

## Background

The disproportionate impact of COVID-19 on ethnic minority communities, including higher rates of diagnosis, hospitalisation and mortality [1], has raised questions about how effectively current UK health policies have incorporated learning from the past few decades about the interplay between ethnicity and health inequalities.

Despite evidence from previous influenza outbreaks that individuals from certain ethnic minority groups experience a higher mortality risk, consideration of ethnicity as a risk factor was perceived to follow media reports of the differential outcomes from COVID-19 infection, rather than emerging from the data [2]. The findings of key national reports on inequalities published during the pandemic widely drew criticism, including from medical and race equality organisations, for, amongst other things, failure to recognise socio-economic determinants of health and failure to recommend action [3]. The potential impact of discriminatory language was highlighted by the change of COVID-19 variant nomenclature by the WHO. Furthermore, prioritisation of digital communications and solutions, may have contributed to widening the existing health equality gap [4]. In this commentary, we place ethnic and digital health inequalities into a broader policy context and highlight areas for action.

## Main text

**Fig. 1:**
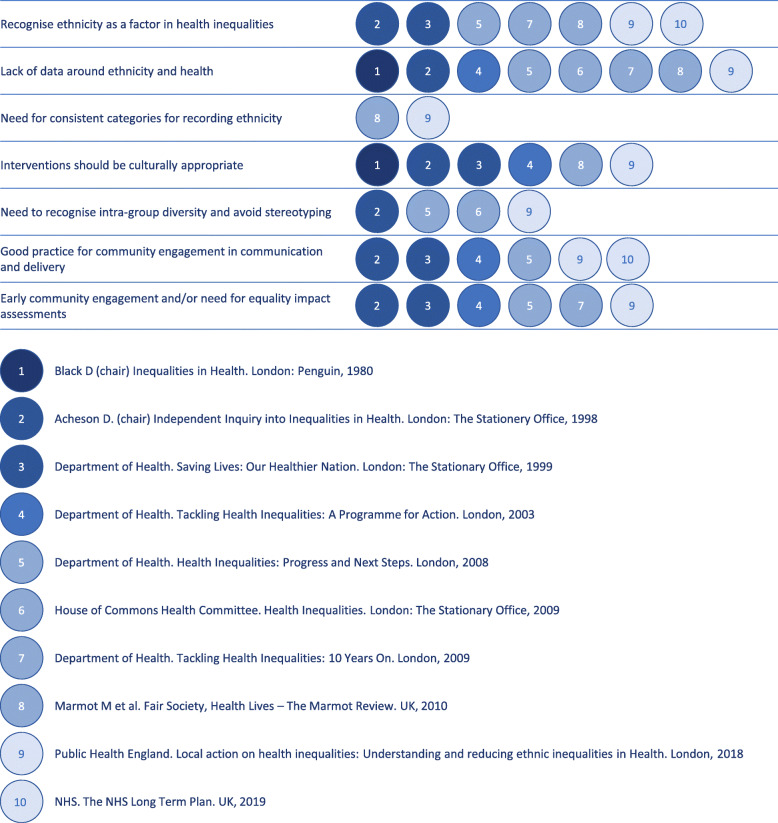
Recommendations for action on health inequalities in England 1980 – 2019. National publications aimed at addressing health inequalities have made similar recommendations over the past four decades. This figure highlights major themes across these publications.

Over the past 20 years there have been a number of national publications about health inequalities, many of which have made similar recommendations for action [see Fig. 1]. For example, both the 1998 Acheson Report, and a 2018 Public Health England report on action on health inequalities identified key areas for improvements including: engagement and development of culturally sensitive interventions which recognise intra-group diversity and avoid stereotyping [5, 6]. It is evident that during this pandemic, these previous ‘lessons learned’ were slow to be applied, and ahead of any public COVID-19 inquiry, it is important to consider how this existing knowledge can be used to address inequalities in the future. Here we highlight three key issues and how they can be addressed: data and measurement, communications, and assessing equality impact.

### 1. Data and measurement of ethnicity

There is a well described gap in ethnicity data within healthcare and routine databases [7], despite statutory requirements for collection, analysis and reporting [6]. There have been increasing concerns that a lack of representativeness in clinical trial recruitment, the data science workforce, and in health data more generally, contributes to “health data poverty”, in which particular individuals, groups, and populations do not benefit equally, or may even be harmed, by a lack of representative data [8]. This was borne out early during the pandemic, with few publications reporting ethnicity disaggregated data, reflecting a lack of data collection [9]. Healthcare, research, and policy spheres must continue to ensure an appropriate balance is found between protecting individual data, and enabling collection and interrogation of data related to social determinants of health inequalities, to identify gaps in knowledge or services.

Linked to this is the need for improved measurement of ethnicity. A key element of this is ensuring the language used to define categories or coding is appropriate. There is recognition that the term ‘BAME’ (Black, Asian and Minority Ethnic) and even broad ethnic categories such as ‘black’ or ‘south Asian’ bring together groups with different influences in terms of culture, religion, and lived experiences. Categories for ethnicity data collected by researchers and the health sector need to be consistent, meaningful and proportionate, and Khunti and colleagues have suggested reflecting the Office for National Statistics coding with a minimum of 5 or 9 categories, for meaningful grouping for data analysis [7].

### 2. Communication with higher risk groups

Strategies for communication during the pandemic have remained largely digital, and slow to deliver inclusivity. The use of digital sources such as Twitter for last minute communication of COVID-19 health messages, the NHS COVID-19 app for contact tracing, and text message for booking vaccinations, is likely to continue to exclude particular groups from receiving key messages, due to variation in digital access, literacy and language barriers. Data from 2018 showed that 10% of the adult population do not use the Internet, with this figure being higher in older adults, women, disabled adults, and the economically non-active [10]; all groups who may be at higher risk of experiencing health inequalities. Furthermore, while overall differences in internet use by ethnicity have narrowed [10], the proportion of adults over 55 of an Asian background who were recent internet users remains significantly lower than for those of a White ethnicity[11]. However, multiple policies have set out examples of good practice, which should be used to avoid patronising approaches, such as: engaging with community leaders, charities and social enterprises with local connections; culturally appropriate messages and communication media; and using co-production approaches [see Fig. 1 for examples].

### 3. Considering the (in)equality impact

‘Digital first’ approaches which do not consider issues of access have the potential to perpetuate or exacerbate existing inequalities. Recommendations from previous reports amount to the need to consider ethnicity and other factors in health inequalities, from the start of any process, be it research, health policy development and implementation, or health care provision. The Public Sector Equality Duty, introduced as part of the Equality Act 2010, aimed to mainstream equality in the activity of public bodies. However, perceptions of outcomes have been mixed, with a concern that organisations have responded with ‘tick box’ exercises to show consideration of equality issues, rather than using equality impact assessments to address equality issues in decision-making, or to reduce or remove inequalities [12]. However, early and meaningful engagement with the relevant groups from the start of any project - for example through representative consultation forums, Public and Patient Involvement, or using co-production approaches - can reduce the risk of unintended consequences, and improve the ability to identify and mitigate inequalities.

## Conclusion

The disproportionate impact of COVID-19 on those groups experiencing greater health inequalities, including those from ethnic minorities, has unfortunately not come as a surprise. There has been a failure to make significant progress on health inequalities, despite a number of national publications and recommendations. The ‘lessons learned’ from the pandemic are not new, and as digital health approaches become more widespread, there is an opportunity to use the greater awareness and ongoing conversations as a catalyst for action to ensure this does not ‘widen the gap’. As we move out of the pandemic and beyond, we have highlighted three areas in which progress should be made: data and measurement, improved communication, and embedded equality impact.

## Data Availability

Not applicable.

## Abbreviations

BAME: Black, Asian and Minority Ethnic
NHS: National Health Service (England)
WHO: World Health Organisation
